# A High Energy Barrier Dy^III^_2_ Single‐Molecule Magnet Supported by a Bulky, Anionic N–O Bridging Ligand

**DOI:** 10.1002/chem.70936

**Published:** 2026-04-01

**Authors:** Alexandros S. Armenis, Concepción Molina‐Jirón, Konstantinos N. Pantelis, Wolfgang Wernsdorfer, Mario Ruben, Eufemio Moreno‐Pineda, Theocharis C. Stamatatos

**Affiliations:** ^1^ Department of Chemistry University of Patras Patras Greece; ^2^ Institute of Quantum Materials and Technologies (IQMT) Karlsruhe Institute of Technology (KIT) Karlsruhe Germany; ^3^ Facultad De Ciencias Naturales, Exactas y Tecnología, Depto. De Bioquímica Universidad De Panamá Panamá Panama; ^4^ Facultad De Ciencias Naturales, Exactas y Tecnología, Grupo de Investigación de Materiales Universidad De Panamá Panamá Panama; ^5^ Physikalisches Institut Karlsruhe Institute of Technology (KIT) Karlsruhe Germany; ^6^ Institute of Nanotechnology (INT) Karlsruhe Institute of Technology (KIT) Karlsruhe Germany; ^7^ Facultad De Ciencias Naturales, Exactas y Tecnología, Depto. De Química‐Física Universidad De Panamá Panamá Panamá

## Abstract

A new dinuclear dysprosium(III) single‐molecule magnet (SMM), [Dy_2_(hynad)_2_(dbm)_4_]·DMF (**1**·DMF), supported by the bulky anionic N–O bridging ligand, N‐hydroxy‐1,8‐naphthalimide (hynadˉ), has been synthesized. The deprotonated hynadˉ ligands enforce a robust and nearly planar {Dy_2_(*µ*‐OR)_2_}^4+^ core, which is surrounded by the sterically demanding dibenzoylmethanoate (dbmˉ) coligands. Each Dy^III^ center adopts a distorted triangular dodecahedral geometry, generating a highly axial crystal field. AC magnetic measurements reveal zero‐field SMM behavior, with slow relaxation of the magnetization persisting up to 16 K, as well as open magnetic hysteresis loops observed by *µ*SQUID magnetometry up to 3.5 K. Analysis of the AC susceptibility data yields an effective energy barrier for magnetization reversal of *U*_eff_ = 171 K. *µ*SQUID studies disclose a characteristic double‐S‐shaped hysteresis, consistent with an antiferromagnetically coupled ground state separated by a small energy gap from a low‐lying ferromagnetic excited state. *Ab initio* calculations confirm the strongly axial *m_J_* = ±15/2 ground Kramers doublets for both Dy^III^ ions, with the magnetic anisotropy axes oriented nearly perpendicular to the {Dy_2_(*µ*‐OR)_2_}^4+^ plane. The combined experimental and theoretical analysis demonstrates that the Dy···Dy interaction is weak and predominantly dipolar in nature, with thermal population of the excited exchange‐coupled state enabling the observed slow magnetic relaxation.

## Graphical abstract

Complex [Dy_2_(hynad)_2_(dbm)_4_] was prepared by the employment of the anionic N–O‐based bridging and chelating *N*‐hydroxy‐1,8‐naphthalimide ligand, featuring a {Dy_2_(*µ*‐OR)_2_}^4+^ core and exhibiting zero‐field SMM behavior with a *U*_eff_ value of 171 K and double‐S‐shaped hysteresis loops, open up to 3.5 K. The weak dipolar Dy···Dy interaction, with thermal population of the excited exchange‐coupled state, enabled the observed slow magnetic relaxation.

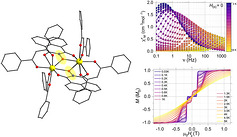

## 1 Introduction

Single‐molecule magnets (SMMs) are molecular systems that exhibit slow relaxation of magnetization arising from intrinsic molecular properties rather than long‐range magnetic ordering [1]. Amongst the known SMMs, lanthanide‐based systems play a central role in molecular magnetism due to their strong spin–orbit coupling and large magnetic anisotropy, with Dy^III^ being particularly effective for achieving high anisotropy [2, 3, 4]. Despite the effectiveness in achieving Dy‐based SMMs, however, quantum tunneling of magnetization (QTM) often limits magnetic bistability in monometallic complexes [5, 6]. To mitigate this effect, dinuclear and multinuclear lanthanide architectures have been widely explored, as magnetic interactions between metal centers can suppress QTM and enhance magnetic relaxation barriers (*U*_eff_) and blocking temperatures (*T*_B_) [7].

In contrast to higher nuclearity clusters, Dy_2_ systems provide well‐defined and experimentally tractable models that enable disentanglement of the respective roles of single‐ion anisotropy, dipolar and/or exchange interactions, governing magnetic relaxation [7, 8]. In such systems, the nature of the bridging ligand (X) plays a decisive role, as it directly determines the Dy···Dy separation, the Dy–X–Dy connectivity, and the symmetry of the local coordination environment [9, 10, 11, 12, 13]. These factors collectively influence both the magnitude of magnetic coupling and the relative orientation of the local magnetic anisotropy axes, which are recognized as key parameters for suppressing efficient QTM and promoting slow magnetic relaxation. Consequently, rational control over the bridging motif in Dy_2_ complexes has emerged as a powerful strategy for probing fundamental magneto‐structural correlations and for optimizing single‐molecule magnet behavior [8, 9, 10, 11, 12, 13].

From a broader perspective, a wide variety of bridging ligands have been employed in the assembly of dinuclear Dy^III^ SMMs, among which oxygen‐donor bridges involving a single bridging oxygen atom (*µ*‐O) are the most prevalent. In particular, alkoxide (ROˉ) [14, 15, 16, 17, 18, 19, 20, 21, 22], phenoxide (R‐PhOˉ) [23, 24, 25, 26, 27, 28, 29, 30, 31, 32], and hydroxide (HOˉ) [33, 34, 35, 36] bridging ligands are among the most commonly encountered motifs, as they readily link two Dy^III^ ions and often enforce relatively short Dy···Dy separations, frequently resulting in a rhombus‐shaped {Dy_2_(*µ*‐O)_2_}^4+^ core. When combined with careful control over the coordination geometry and the imposition of strongly axial ligand fields at each Dy^III^ ion, several oxygen‐bridged Dy_2_ SMMs have been reported to exhibit large effective energy barriers (*U*_eff_), in some cases reaching several hundred Kelvin [14, 15, 16, 17, 18, 19, 20, 21, 22, 23, 24, 25, 26, 27, 28, 29, 30, 31, 32, 33, 34, 35, 36]. Alternative strategies have focused on radical‐bridged Dy_2_ systems, in which inorganic (N_2_^3−^) or organic ligand radicals (e.g., tetrazine, tetrapyridylpyrazine, bipyrimidine, bisbenzimidazole, tetraazanaphthalene, fluoflavine) mediate magnetic exchange and introduce additional spin degrees of freedom [37, 38, 39, 40, 41, 42, 43, 44, 45]. These 2p‐4f molecular systems can lead to enhanced exchange coupling between 4f‐metal centers, mitigating the detrimental zero‐field QTM relaxation process, thus yielding large coercive field values and higher magnetization blocking temperatures. Simpler halide bridging ligands (e.g., chloride, bromide, iodide) have also been explored, offering weakly donating, monoatomic connections that can preserve axial crystal fields and reduce perturbations to the Dy^III^ anisotropy, affording ‐in some cases‐ remarkably high effective barriers, in the range of 10^3^ Kelvin [46, 47, 48, 49]. It is worth mentioning that a recently reported triply iodide‐bridged dinuclear organometallic Dy_2_ complex represents a rare mixed‐valence system, where *µ*_3_‐iodide ligation and direct Dy···Dy interactions give rise to exceptionally strong magnetic coupling and ultrahard single‐molecule magnet behavior resulting in record coercive fields among all known SMMs to date [50].

Among the various bridging motifs explored in Dy_2_ single‐molecule magnets, N–O‐based ligands have attracted considerable attention due to their ability to efficiently mediate magnetic exchange between highly anisotropic Dy^III^ centers. In particular, neutral pyridine N‐oxides are now well established as effective *µ*‐OR bridges, giving rise to a wide range of dinuclear Dy^III^ complexes with tunable magnetic behavior [51, 52, 53, 54, 55, 56, 57, 58, 59, 60, 61, 62, 63, 64, 65, 66, 67, 68, 69, 70, 71]. Significant progress in this area has demonstrated that simple pyridine‐N‐oxide bridges, when combined with *β*‐diketonate ligands, can generate pseudo‐axial DyO_8_ coordination environments that efficiently suppress QTM and enable zero‐field SMM behavior [53, 58, 60, 65, 66, 70]. This foundational structural motif has since been expanded through systematic modification of the N‐oxide bridges, including substituted pyridine N‐oxides, pyrazine‐N‐oxide, bipyridine N,N′‐dioxides, and other heterocyclic or ditopic N‐oxide ligands, most commonly in combination with hfacˉ (hexafluoroacetylacetonate), ttaˉ (thenoyltrifluoroacetonate), or related ancillary *β*‐diketonate ligands [51, 52, 53, 54, 55, 56, 57, 58, 59, 60, 61, 62, 63, 64, 65, 66, 67, 68, 69, 70, 71]. In contrast, anionic N–O‐based ligands bearing a localized negative charge on the oxygen atom capable of supporting dinuclear Dy^III^ SMMs remain comparatively rare and underexplored [72, 73].

**SCHEME 1:**
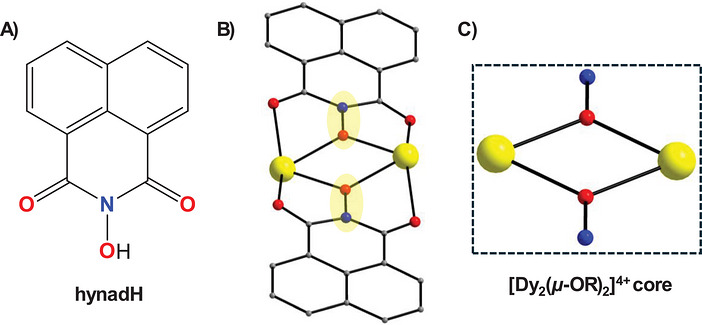
(A) Chemical structure of the *N*‐hydroxy‐1,8‐naphthalimide (hynadH) ligand, (B) bridging and chelating coordination mode of hynadˉ forming the robust [Dy_2_(*µ*‐OR)_2_]^4+^ core, and (C) rhombus‐shaped [Dy_2_(*µ*‐OR)_2_]^4+^ core resulting from the deprotonated N–O^−^ groups of hynadˉ. Color code: Dy^III^, yellow; O, red; N, blue; C, dark gray.

A notable contribution to this area was recently reported by some of us, by introducing the organic ligand *N*‐hydroxy‐1,8‐naphthalimide (hynadH, Scheme 1A), which upon deprotonation can act as a new anionic N–O‐based chelating and bridging ligand in dysprosium chemistry (Scheme 1B) [74]. In this reported work, a family of dinuclear complexes featuring a nearly planar {Dy_2_(*µ*‐OR)_2_}^4+^ rhombus‐shaped core was isolated (Scheme 1C), demonstrating that the deprotonated R_2_N–Oˉ moiety can act as an efficient monoatomic bridge between two Dy^III^ centers while simultaneously chelating each metal ion. These studies firmly established hynadˉ as a rare example of an anionic N–O‐based ligand capable of stabilizing well‐defined {Dy_2_(*µ*‐OR)_2_} metal cores exhibiting SMM behavior [74]. Building upon this foundation, we further reported the structurally related dinuclear complex [Dy_2_(hynad)_2_(dpm)_4_], where dpmˉ (dipivaloylmethanoate) ligands occupy the peripheral coordination sites while preserving the central {Dy_2_(*µ*‐OR)_2_}^4+^ core enforced by the deprotonated *N*‐hydroxy‐1,8‐naphthalimide bridges [75]. Notably, this compound exhibited pronounced SMM behavior, with a *U*_eff_ that was the highest reported to date among Dy_2_ complexes constructed with the hynadˉ bridging ligand. This result demonstrated not only the robustness of the {Dy_2_(*µ*‐OR)_2_}^4+^ motif supported by hynadˉ, but also its capacity to promote favorable magnetic anisotropy and slow relaxation dynamics when combined with suitably chosen ancillary *β*‐diketonate ligands.

In this context, the present study extends this chemistry through the synthesis and characterization of the closely related complex [Dy_2_(hynad)_2_(dbm)_4_]·DMF (**1**·DMF), in which the peripheral dipivaloylmethanoate groups are replaced by the sterically demanding dibenzoylmethanoate ligands. Despite this substitution, the deprotonated *N*‐hydroxy‐1,8‐naphthalimide ligands retain their *µ*‐O bridging role, giving rise once again to a nearly planar [Dy_2_(*µ*‐OR)_2_]^4+^ core. To gain a comprehensive understanding of the magnetic properties arising from this robust [Dy_2_(*µ*‐OR)_2_]^4+^ core, we have undertaken a combined experimental and theoretical investigation of the aforementioned complex. In the present work, the magnetic behavior was examined using DC magnetometry to probe the static magnetic response and magnetic anisotropy, AC susceptibility measurements to characterize the slow relaxation of the magnetization and SMM behavior, and *µ*SQUID magnetometry to directly assess magnetic hysteresis down to the sub‐Kelvin temperature regime. In parallel, *ab initio* calculations were performed to elucidate the electronic structure of the Dy^III^ centers, determine the orientation of the local magnetic anisotropy axes, and evaluate the nature and magnitude of the magnetic interactions transmitted through the [Dy_2_(*µ*‐OR)_2_]^4+^ core. Hence, this combined approach allows a detailed structure‐property correlation to be established and provides insight into the role of the R_2_N–Oˉ bridges in governing both the single‐ion anisotropy and the intramolecular Dy···Dy magnetic coupling.

## 2 Results and Discussion

### 2.1 Synthetic Comments

Following our previous works on dinuclear dysprosium complexes supported by the anionic N–O‐based ligand hynad‐, in which systematic modulation of peripheral coordination environments was achieved through variation of nitrate and *β*‐diketonate ligands, we sought to further expand this family by introducing sterically more demanding *β*‐diketonates. In analogy with the reported syntheses of [Dy_2_(hynad)_2_(dpm)_4_] and related systems [74, 75], the reaction between Dy(dbm)_3_·H_2_O and hynadH in the presence of Et_3_N was carried out using a 1:1:1 molar ratio in a CH_2_Cl_2_/DMF solvent mixture (5:1, v/v). Under these mild basic conditions, deprotonation of hynadH promotes formation of the characteristic double N–O‐bridged {Dy_2_(*µ*‐OR)_2_}^4+^ core, while the bulky dibenzoylmethanato ligands occupy the remaining coordination sites and impose increased steric pressure on the dinuclear unit. This approach afforded well‐shaped block‐like yellow crystals of the dinuclear complex [Dy_2_(hynad)_2_(dbm)_4_]·DMF (**1**·DMF), suitable for single‐crystal X‐ray diffraction. Notably, despite replacement of the less bulky dpmˉ chelates [74, 75] with dbmˉ, the robust and nearly planar {Dy_2_(hynad)_2_}^4+^ core is retained, highlighting the structural directing role of the hynadˉ ligand and its ability to stabilize dinuclear metallic assemblies across a range of different ancillary ligand environments.

### 2.2 Description of Structure

**FIGURE 1:**
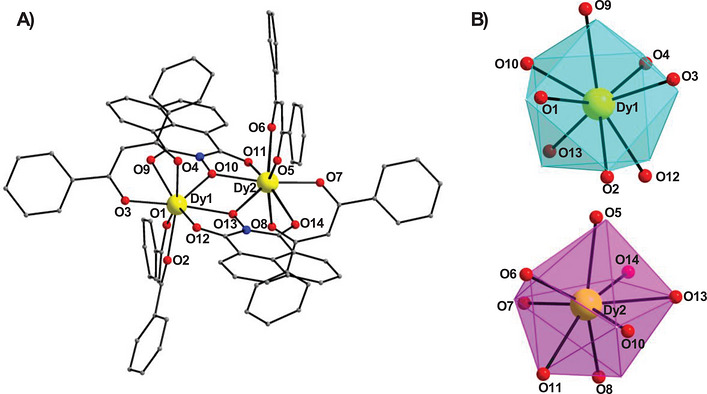
(A) Labeled representation of the crystal structure of complex **1**·DMF, and (B) triangular dodecahedral coordination polyhedra of the crystallographically independent Dy^III^ metal centers in complex **1**·DMF. The smaller white spheres define the vertices of the corresponding ideal polyhedron. All H atoms and the DMF lattice solvent are omitted for clarity. Color code: Dy^III^, yellow; O, red; N, blue; C, dark gray.

Crystallographic details and selected interatomic distances and angles for compound **1**·DMF are listed in Tables S1 and S2, respectively. For the sake of brevity, only the important metrical parameters will be discussed in the main text. Single‐crystal X‐ray diffraction analysis reveals that compound **1**·DMF is a dinuclear Dy^III^ complex with the molecular formula [Dy_2_(hynad)_2_(dbm)_4_] (Figure 1A). Complex **1**·DMF crystallizes in the trigonal space group *R*3*c*, and its crystal packing is stabilized by one lattice DMF molecule per dinuclear unit. The asymmetric unit consists of a dinuclear Dy^III^ complex containing two crystallographically unique metal centers (Dy1 and Dy2). These metal centers are doubly bridged by the R_2_N–Oˉ groups (through O10 and O13 atoms) of two deprotonated hynadˉ ligands, yielding a rhombus‐shaped {Dy_2_(*µ*‐OR)_2_}^4+^ core. The {Dy_2_(*µ*‐OR)_2_}^4+^ core, which consists of two tridentate chelating and bridging (η^1^:η^2^:η^1^:*µ*) hynadˉ ligands arranged opposite to each other, is further stabilized by the formation of two five‐membered chelate rings from each hynadˉ ligand, involving the two carbonyl oxygen atoms (O9, O11 and O12, O14) and the oxygen atoms (O10 and O13) of the R_2_N–Oˉ groups. Peripheral coordination in complex **1**·DMF arises from four O,O′‐chelating dbmˉ ligands (O1, O2, O3, O4 for Dy1 and O5, O6, O7, O8 for Dy2) located above and below the plane of the central metal core, resulting in a DyO_8_ coordination environment for each metal ion. Both Dy^III^ ions are thus 8‐coordinate, and according to the SHAPE program, they adopt distorted triangular dodecahedral coordination geometries (Figure 1B) with similar CShM values (3.38 for Dy1 and 3.22 for Dy2, Table S3). The similarity in the coordination polyhedra is further confirmed by the almost identical Dy–O bond distances and O–Dy–O bond angles between Dy1 and Dy2 units. The intradimer Dy1···Dy2 distance is 3.993(8) Å while the Dy1–O10–Dy2 and Dy1–O13–Dy2 angles are 118.5(1)° and 118.1(1)°, respectively. Likewise, the shortest intermolecular Dy···Dy distance resulting from two adjacent dimeric Dy_2_ complexes is 9.067(5) Å. Interestingly, the Dy_2_ complexes (Figure S1a) are densely packed and interconnected through extensive *π*–*π* stacking interactions between the naphthalene backbone of the hynadˉ ligands and the twisted phenyl rings of the dbmˉ ligands of neighboring Dy_2_ units. The corresponding centroid‐to‐centroid distances are 3.643 and 3.662 Å (Figure S2). Viewed along the crystallographic *c*‐axis, the dinuclear Dy_2_ units adopt a cyclic supramolecular arrangement in which the phenyl rings of the peripheral dbmˉ ligands are oriented toward the interior of the assembly, giving rise to star‐like packing motifs stabilized by *π*–*π* interactions (Figure S1b).

### 2.3 Magnetic Studies

#### 2.3.1 Static Magnetic Properties

**FIGURE 2:**
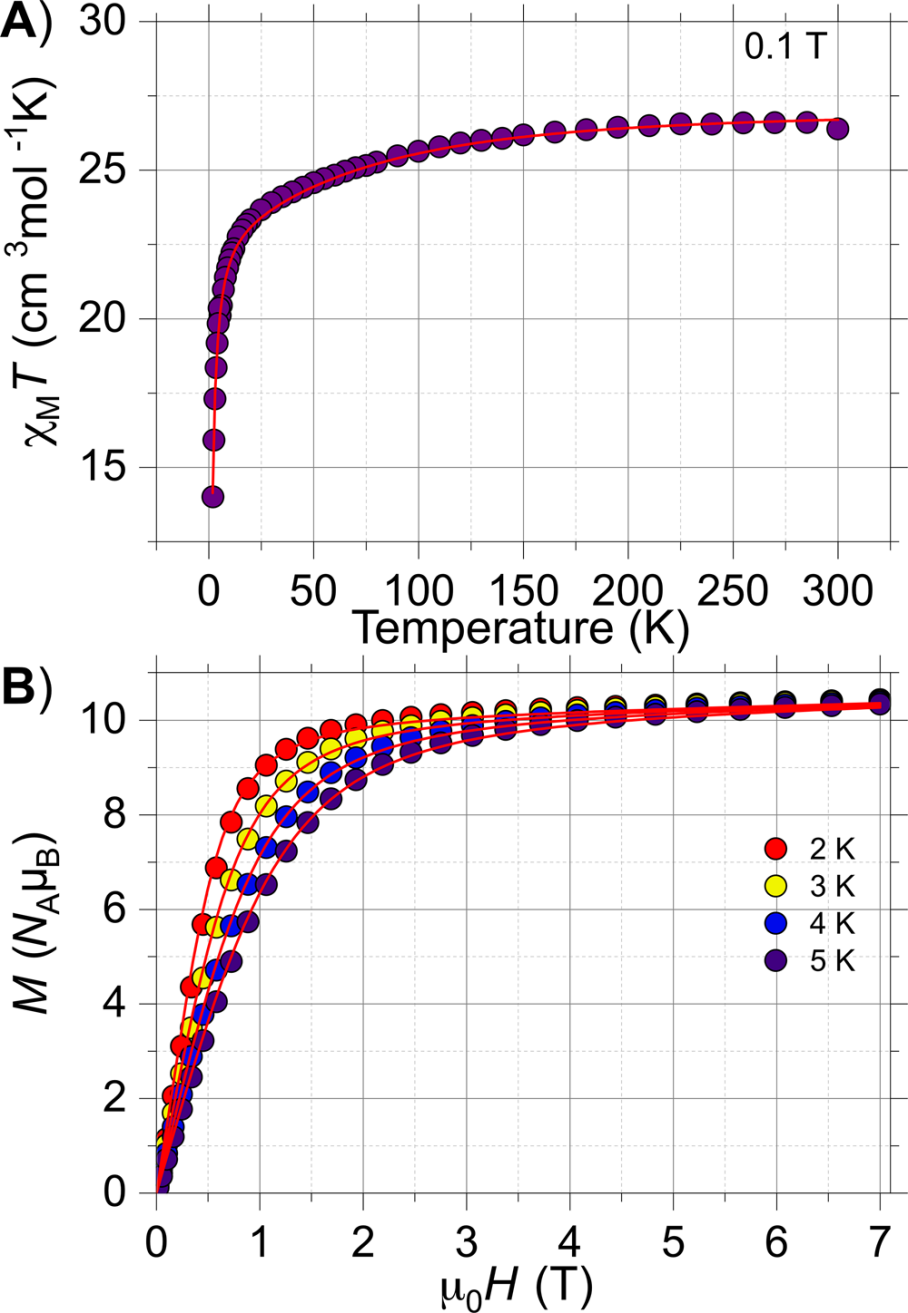
Temperature dependence of the *χ*_M_ *T* product at 0.1 T static DC field (A) and field (*H*) dependence of the magnetization (*M*) at four different low temperatures (B) for complex **1**. Experimental data are shown as filled circles. Solid lines are *χ*_M_ *T*(*T*) and *M*(*H*) traces obtained employing the crystal field parameters from CASSCF calculations (see the text for details).

Direct‐current (DC) magnetic susceptibility measurements were performed on a microcrystalline sample of **1** over the temperature range 1.9–300 K under an applied magnetic field of 0.1 T (Figure 2A). The chemical purity of the sample used for the magnetic studies was confirmed by elemental analysis, allowing accurate determination of its exact molecular weight. The room temperature *χ*_M_ *T* value of 27.8 cm^3^ mol^−1^ K is very close to the theoretical value of 28.3 cm^3^ mol^−1^ K for two noninteracting Dy^III^ ions (^6^H_15/2_, *S* = 5/2, *L* = 5 and *g* = 4/ 3). Upon cooling, the *χ*_M_ *T* value decreases gradually down to 8 K, and it then drops abruptly before reaching a value of 14.8 cm^3^ mol^−1^ K at the lowest measured temperature (2 K). The rapid decline of *χ*_M_ *T* at low temperatures is attributed to the depopulation of the *m_J_* sublevels of the ground *J* state, and possibly to the presence of weak antiferromagnetic interactions between the two Dy^III^ ions.

The field (*H*) dependence of the magnetization (*M*) for complex **1** measured at 2, 3, 4, and 5 K is shown in Figure 2B. The magnetization increases rapidly at low applied fields but does not reach full saturation even at the maximum applied field of 7 T, indicating the presence of significant magnetic anisotropy and/or low‐lying excited states. At 2 K, the magnetization tends toward saturation above approximately 2 T, reaching a value of 10.4 *N*µ_B_ at 7 T. This value is significantly lower than the expected saturation magnetization (*M*_sat_) value for two noninteracting Dy^III^ ions (*M*_sat_/*N*µ_B_ = 20 *Nµ*_B_), reflecting strong magnetic anisotropy and crystal‐field effects.

#### 2.3.2 Dynamic Magnetic Properties

**FIGURE 3:**
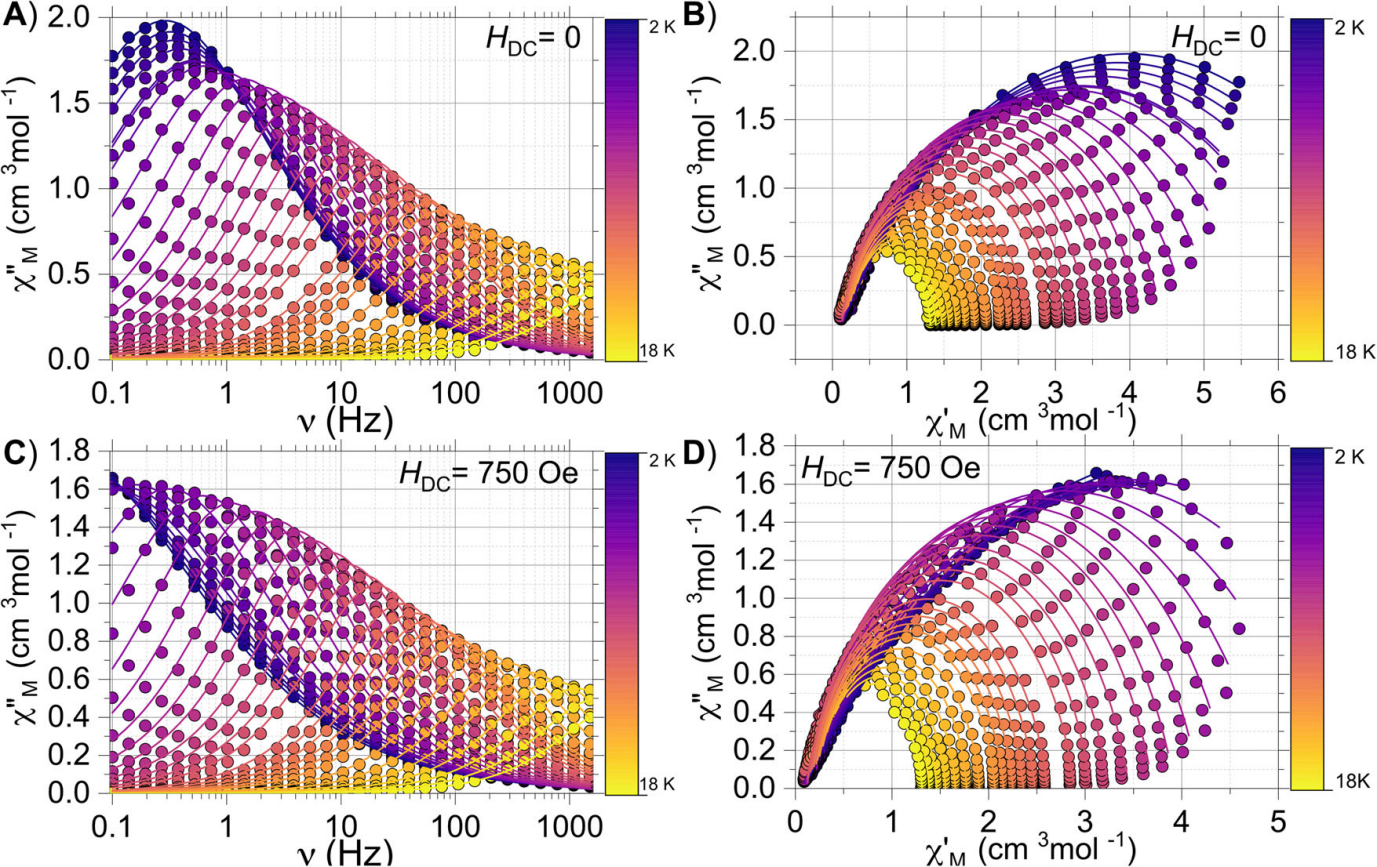
Frequency‐dependence of the out‐of‐phase (*χ*_M_ *″*) AC magnetic susceptibility over the temperature range 2‐18 K, at zero DC field (A), and under an applied field of 750 Oe (C) for complex **1**. Cole‐Cole plots for complex **1** obtained using the AC susceptibility data at zero DC field (B), and under an applied field of 750 Oe (D). Solid lines represent the best fit to a generalized Debye model.

To investigate the magnetization relaxation dynamics of complex **1** and to validate its SMM properties, alternating current (AC) magnetic susceptibility measurements were initially performed at zero applied DC field in the temperature range of 1.8–18 K, under an AC field of 3.0 Oe, with frequencies ranging from 0.1 to 1512 Hz. The pronounced frequency‐ and temperature‐dependent behavior of both the in‐phase (*χ*_M_′) and out‐of‐phase (*χ*_M_ *″*) magnetic susceptibilities unambiguously confirms SMM behavior in the absence of an applied DC field for complex **1** (Figures 3 and S3–S5). Interestingly, the temperature dependence of the out‐of‐phase AC magnetic susceptibility, *χ*_M_ *″*(*T*), exhibits a well‐resolved maximum at 16 K at the highest frequency of 1512 Hz (Figure S5A). Frequency‐dependent magnetic susceptibility (*χ*_M_ *″*(*ν*)) studies revealed a maximum centered at 0.2 Hz at the lowest temperature of 2 K. This maximum remains essentially temperature independent upon increasing the temperature up to 4 K, due to the dominant QTM relaxation. Above 4 K, the peak maxima become distinctly temperature dependent, progressively shifting toward higher frequencies with increasing temperature, due to the onset of phonon‐assisted relaxation pathways (Raman and Orbach mechanisms). In addition, the shapes of the Cole‐Cole plots deviate from the typical semicircular ones, yielding *α* values in the range of 0.40–0.05 (Table S4), indicative of a wide distribution of relaxation times, which is consistent with the presence of multiple relaxation processes. Furthermore, the *χ*_M_′*T* value (*χ*_M_′ being the in‐phase AC susceptibility) at its plateau temperature region is ∼25 cm^3^ K mol^−1^ (Figure S3B), which is very close to the expected value (∼27 cm^3^ K mol^−1^) for randomly oriented crystals of a molecule with two strongly anisotropic Dy^III^ ions, each with a *m_J_* = ± 15/2 Ising ground Kramers’ doublet [76].

**FIGURE 4:**
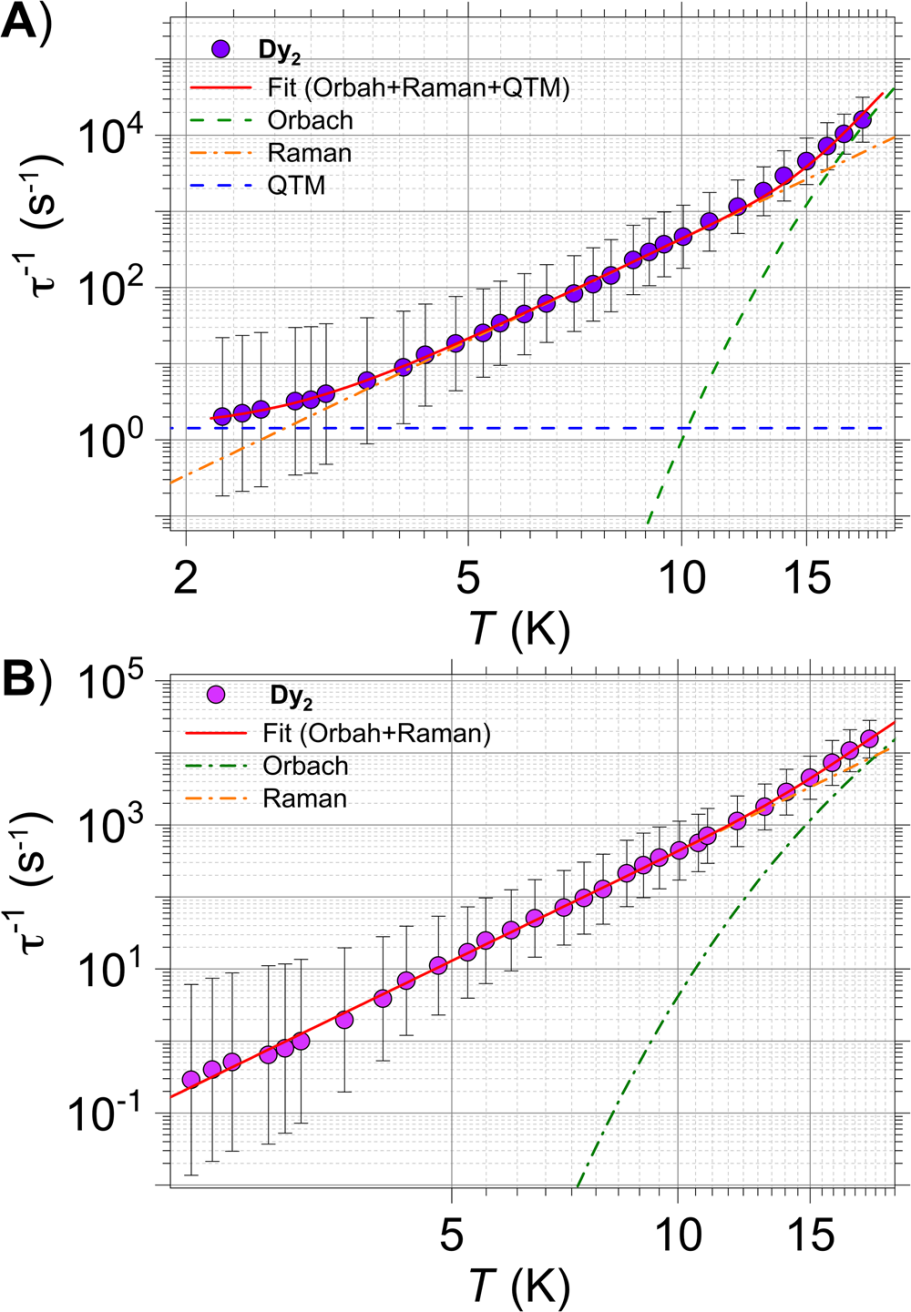
Temperature dependence of the relaxation rates (*τ* ^−1^) at zero DC field (A), and under an applied DC field of 750 Oe (B), fitted by implementing Equations (1) and (2), respectively. The solid circles correspond to the experimental data, while the continuous red solid line represents the best fit obtained from the above equations. The individual contribution of each relaxation process is illustrated as dashed lines, and error bars represent the standard deviations in the relaxation rates obtained from the fitting of the AC susceptibility data.

The relaxation times (*τ*) can be extracted from the AC magnetic susceptibility data by simultaneously fitting *χ*_M_′(*ν*) and *χ*_M_′′(*ν*) in the range of 2–18 K employing a generalised Debye model. The temperature dependence of the relaxation times at zero DC field can be fitted employing the QTM, Raman and Orbach processes by using the following Equation (1) [77, 78]:

(1) $$\tau^{-1}={\tau_{0}}^{-1}\exp\left(-U_{\mathrm{eff}}/k_{B}T\right)+CT^{n}+{\tau_{\mathrm{QTM}}}^{-1}$$

where *τ* ^−1^ defines the relaxation rate, and the first term corresponds to the Orbach process in which *τ*_0_ is the pre‐exponential factor, *k*_B_ *T* is the thermal energy, and *U*_eff_ is the effective energy barrier for the magnetization reversal. In addition, *C* and *n* are parameters of the Raman relaxation, and *τ*_QTM_^−1^ is the rate of the QTM process. The best‐fit of the data (solid red line in Figure 4A ) yielded the values: *τ*_0_ = 6.8(3) × 10^−9^ s, *U*_eff_ = 119(1) cm^−1^ [171(1) K], *C* = 1.36(4) × 10^−2^ s^−1^ K^−^ *^n^*, *n* = 4.52(3), and *τ*_QTM_ = 0.65(2) s. The *U*_eff_ value of complex **1** is of similar magnitude with those reported for the highest performing pyridine N‐oxide bridged Dy_2_ SMMs supported by *β*‐diketonate ligands (Table S5).

**FIGURE 5:**
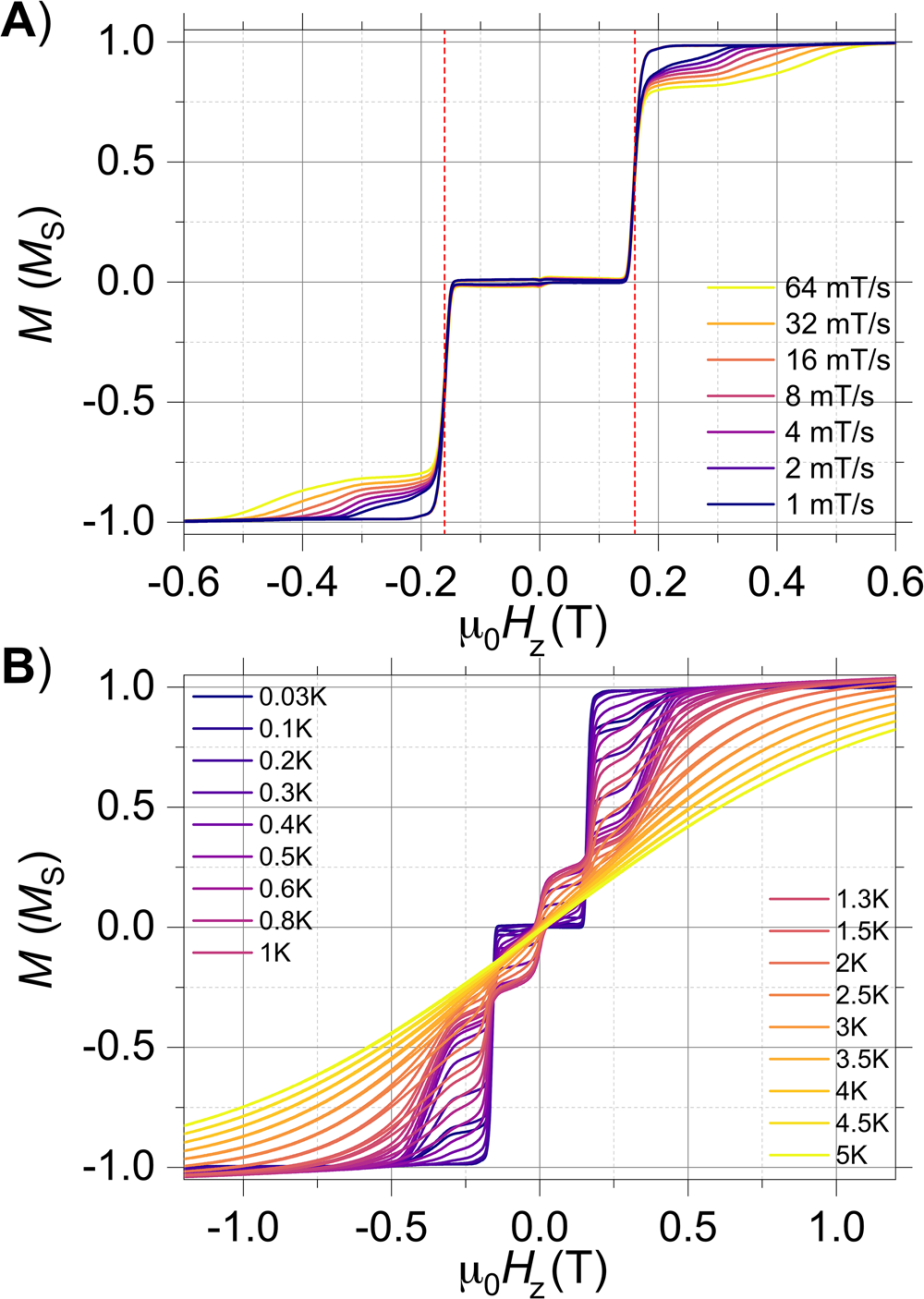
µSQUID hysteresis loops: (A) Sweep‐rate dependence of the *M*(*H*) data for Dy_2_ recorded at 30 mK. The inflection points at ±0.18 T (red dotted lines), and the nearly flat region indicate an antiferromagnetic ground state and the presence of a mean exchange field operating between the Dy^III^ ions. (B) Temperature dependence of the *M*(*H*) loops measured between 30 mK and 3.5 K at a field sweep rate of 16 mT/s, leading to open hysteresis loops and demonstrating the slow magnetic relaxation characteristics of complex **1**·DMF.

To reduce the contribution of QTM on the magnetization relaxation, AC magnetic susceptibility measurements were performed under an applied optimal DC field of 750 Oe (see Figures S6 and S7). As a result, the low‐temperature tails observed in the *χ*_M_″(*T*) plots were strongly suppressed and ultimately vanished (Figure S5), indicating efficient quenching of QTM. The removal of these features has contributed toward the slowing of the relaxation process and the shifting of the *χ*_M_″ peak maxima outside the experimental frequency window (Figure 3C). Under the applied DC field of 750 Oe, the *χ*_M_″(*v*) maxima measured at different temperatures have become more clearly resolved, leading to improved separation of the peaks compared to the zero‐field AC data (Figures 3A and 3C). The shape of the Cole‐Cole plots in the presence of a DC field is more circular and less asymmetric than in zero field, especially at low temperatures, yet denoting a wide distribution of relaxation times, as evidenced by the *α* values in Table S6. In this case, the temperature dependence of relaxation times was modelled by excluding the QTM term (solid red line in Figure 4B), using the following Equation (2):

(2) $$\tau^{-1}={\tau_{0}}^{-1}\exp\left(-U_{\mathrm{eff}}/k_{B}T\right)+CT^{n}$$

The relaxation parameters obtained under an applied DC field of 750 Oe are: *τ*_0_ = 1.1(1) × 10^−8^ s, *U*_eff_ = 117(4) cm^−1^ [169(6) K], *C* = 3.8(4) × 10^−2^ s^−1^ K^−^ *^n^*, *n* = 5.0(2). Although the application of an external DC field reduces QTM at the lowest temperatures, it does not lead to any increase in the energy barrier for magnetization reversal. The obtained Raman parameters, *C* and *n*, are within the expected range for Dy^III^‐based SMMs [14, 15, 16, 17, 18, 19, 20, 21, 22, 23, 24, 25, 26, 27, 28, 29, 30, 31, 32, 33, 34, 35, 36, 37, 38, 39, 40, 41, 42, 43, 44, 45, 46, 47, 48, 49, 50]. The observed lower *n* values (*n* = 9 for Kramers ions) are possibly attributed to the presence of low‐energy phonon modes and/or slight deviations from ideal Raman relaxation [79, 80, 81]. Intriguingly, the *τ*(*H*) study (Figure S7), besides providing the field at which relaxation is slowest, also shows fast relaxation occurring at 0.18 T.

### 2.4 µSQUID Hysteresis Studies

Confirmation of the SMM characteristics of complex **1** was obtained by investigating its magnetic hysteresis behavior. For this, a sub‐kelvin investigation was carried out employing highly sensitive µSQUID arrays. The µSQUID measurements were performed on single crystals of complex **1**·DMF with the field applied along the easy axis of the crystal, employing the transverse field method [82]. The studies comprise field sweep rates from 64 mT/s down to 1 mT/s and temperatures ranging from 30 mK up to 5 K.

µSQUID measurements revealed open hysteresis loops for the Dy_2_ complex at the lowest measured temperatures (30 mK) that remain open until 3.5 K (Figure 5B), while the sweep‐rate‐dependent loops highlight the expected behavior of SMMs (Figure 5A). Moreover, at the lowest temperature, the dimeric nature of the complex is readily visible, as shown by the double‐S‐shaped hysteresis loops, typical of an antiferromagnetically coupled Dy_2_ SMM [26, 30, 83, 84, 85, 86]. At high negative field and 30 mK temperature, the ferromagnetically coupled excited state is populated. Upon decreasing the field, a crossing with the antiferromagnetically coupled ground state occurs at ‐0.18 T. Between the ±0.18 field, nearly no magnetic moment is observed, as expected for a purely diamagnetic ground state. Above +0.18 T, a crossing between the antiferromagnetic ground state and the excited ferromagnetic state occurs, leading to the progressive population of the ferromagnetic ground state (Figure 5A). The fast relaxation observed in the *τ*(*H*) dependence from the AC susceptibility measurements can therefore be attributed to enhanced QTM at the level crossing between the exchange‐coupled ground and first excited states (Figure S7).

Additionally, within ±0.18 mT, the magnetic moment is nearly zero at the lowest temperatures due to the diamagnetic ground state; upon increasing temperature, thermal population of the ferromagnetic excited state leads to a progressive increase in the magnetization in this field region, as evidenced by the comparison between the 30 mK hysteresis loops and those recorded at higher temperatures (Figure 5B). The inflection points observed in the µSQUID loops allow the determination of the mean exchange field (*H*_ex_), leading to an effective exchange constant between the Ising spins of the Dy^III^ ions according to the following Equation (3):

(3) $$H_{ex}=\frac{-2\;J_{ex}m_{J}}{g_{J}\mu_{B}}$$

where *m_J_* = 15/2 and *g_J_* = 4/3 [30, 87]. This leads to an effective exchange constant of *J*_ex_ = ‐0.0066 cm^−1^ (‐9.5 mK). The determined *J*_ex_ of ‐9.5 mK is close to the value obtained from a purely point dipolar approximation: *D*_dip_ = ‐5 mK for a Dy···Dy distance of 3.993(8) Å, suggesting that the interaction between the Dy^III^ atoms is most likely purely of dipolar origin, although exchange cannot completely be excluded [30].

### 2.5 Theoretical Calculations

**FIGURE 6:**
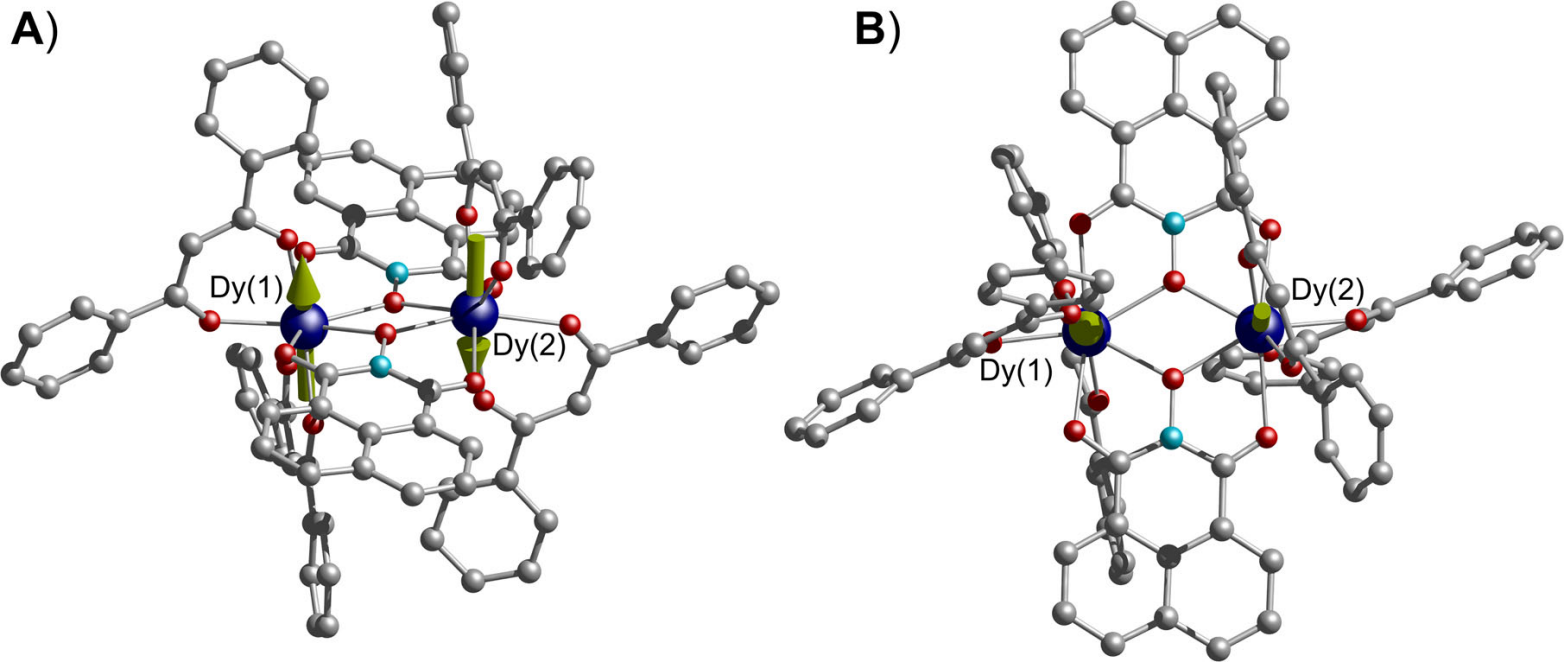
Side (A) and top (B) views of the calculated orientations of the principal axes (green arrows) of the *g*‐tensors for the ground Kramers doublets of Dy1 and Dy2 in complex **1**. Color code: Dy, blue; C, gray; N, cyan; O, red. Hydrogens were omitted for clarity.

To comprehend the magnetic characteristics of complex **1_,_** we carried out *ab initio* calculations using the CASSCF/SO‐RASSI/SINGLE_ANISO approach (see the *ab initio* section for details). For this purpose, the crystal structure of the Dy_2_ complex was employed without further optimization. The energies of the low‐lying Kramers doublets (KDs), the main components of the *g*‐tensor, along with the *m_J_* components for each KD for Dy1 and Dy2, are shown in Tables S7 and S8, respectively. Inspection of the low‐lying electronic structure of both Dy^III^ ions revealed highly axial *g*‐values in the ground ±*m_J_* states approaching the Ising limit, i.e., *g*_x_ = *g*_y_ ∼ 0 and *g*_z_ ∼ 20. The ground KDs for both Dy^III^ ions at the Dy_2_ complex are predominantly comprised of a *m_J_* = ±15/2 doublet ( > 98%), while the first excited states are found to be highly mixed. For Dy1, the first excited state is found to lie at 118 cm^−1^, while for Dy2, the excited state lies at 84 cm^−1^. The easy axes of magnetization lie nearly perpendicular to the plane defined by both Dy^III^ ions and the R_2_N–Oˉ groups from the hynadˉ ligands, corresponding to the central {Dy_2_(*µ*‐OR)_2_}^4+^ core, as depicted in Figure 6. Specifically, the magnetic anisotropy vectors of Dy1 and Dy2 are aligned along the axial O4 and O8 atoms of the dbmˉ ligands, respectively. This alignment arises from the relatively short Dy–O bond distances, which dominate the local crystal field environment [88].

Although the CASSCF results indicate that the single‐ion characteristics for each Dy^III^ resulting from the crystal field environment are highly anisotropic, with a relatively large energy separation between the ground and first excited ±*m_J_* states, the magnetic behavior must also take into account the presence of interactions operating between the Dy^III^ atoms. With the knowledge of the crystal field parameters from CASSCF (Table S9), it is possible to evaluate whether the downturn in the *χ*_M_ *T*(*T*) data corresponds to the two interacting Dy^III^ ions. For this, we employed the Lines model by simultaneously fitting the *χ*_M_ *T*(*T*) and *M*(*H*) data sets [89]. The Lines model employs an isotropic exchange between the spin component of the angular momenta (*S* = 5/2 for Dy^III^) and the crystal field parameters obtained from the CASSCF calculations. The Hamiltonian is described by the following Equation (4):

(4) $$H_{\mathrm{Dy}}^{i}=H_{\mathrm{lf}}^{i}+g_{J}\mu_{0}\mu_{B}\left({\hat{J}}_{\mathrm{Dy}1}+{\hat{J}}_{\mathrm{Dy}2}\right)H_{z}-2J_{\mathrm{Lines}}^{i}\left({\hat{S}}_{\mathrm{Dy}1}\cdot {\hat{S}}_{\mathrm{Dy}2}\right)$$

where $H_{lf}^{i}=\sum_{k=2,4,6,-k\leq q\leq k}B_{k}^{q}O_{k}^{q}$ is the ligand field Hamiltonian expressed in the Steven's operators, $O_{k}^{q}\;$ are the Stevens operators, and $B_{k}^{q}$ are the ligand field parameters obtained from CASSCF calculations. In addition, ${\hat{J}}_{Dy}$ and ${\hat{S}}_{Dy}$ are the spin‐orbit and spin‐only states for each Dy^III^ atom, respectively. Simultaneous fit of the *χ*_M_ *T*(*T*) and *M*(*H*) data has yielded a *J*_Lines_ = ‐0.0046(1) cm^−1^ (6.6 mK) (see solid lines in Figure 2) [90]. Considering the interaction operating within the dimer, the ground doublet state is a singlet, with a separation of ∼1 cm^−1^ from the first excited state multiplet. Considering that the separation is small and the operating temperature of our experiments ( > 2K), the excited state is readily populated, hence the SMM character in this system arises from the first excited multiplet state. Above this state, the next excited multiplet lies at ∼86 cm^−1^, which coincidentally is very close to the *U*_eff_ determined from the AC studies, thus relaxation occurs via this state.

**FIGURE 7:**
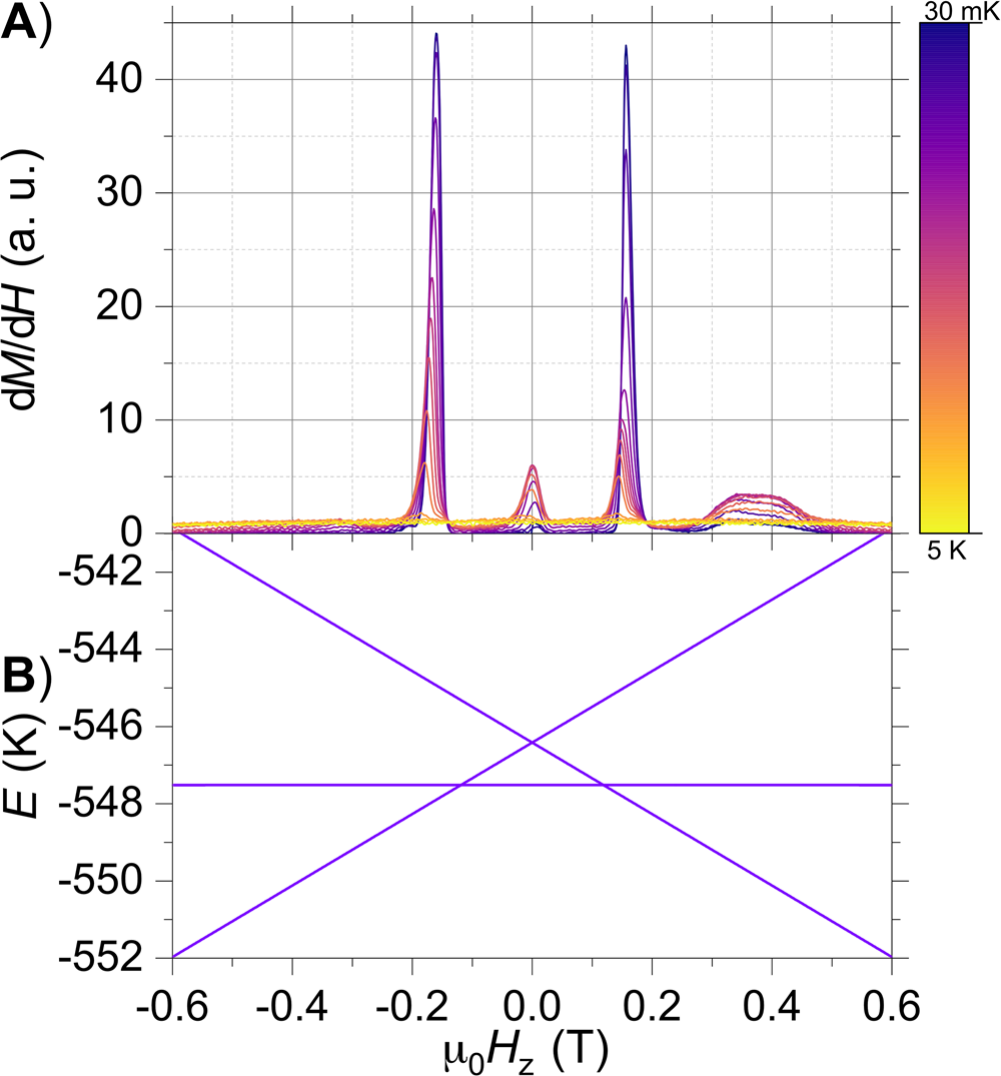
(A) Derivative of the temperature‐dependent *M*(*H*) hysteresis loops at 16 mT/s and (B) Zeeman diagram obtained employing the crystal field parameters from CASSCF and the *J*_Lines_ obtained from fitting the *χ*_M_ *T*(*T*) and *M*(*H*) data.

Although a direct comparison of the *J*_Lines_ and the *J*_ex_ is not possible, projecting the *J*_Lines_ for a *S* = 5/2 into a *J* = 15/2 state leads to a value of *J*_Lines_ (15/2) = ‐0.005 cm^−1^ (‐7 mK), close to the experimentally obtained *J*_ex_ of ‐9.5 mK. This is readily visible when comparing the derivative of the temperature dependence of the *M*(*H*) loops and the Zeeman diagram employing the Lines model (Figure 7). As shown in Figure 7, the extremely narrow remanence is indicative of an antiferromagnetic ground state persisting within ±0.18 T, as confirmed by the first‐derivative magnetization curves (d*M*/d*H*). In this field region, the magnetization is essentially zero. The sharp peaks observed in the d*M*/d*H* plot at ±0.18 T are nearly coincident with the predicted Zeeman crossings (red dotted lines in Figure 7) between the antiferromagnetic ground state (horizontal line) and the first excited ferromagnetic state. Beyond these field limits, the thermal population of magnetic states gives rise to SMM behavior, with switching fields that closely match the computationally determined intramolecular magnetic field (0.12 T) from the Zeeman diagram. The small peak observed at zero field in the d*M*/d*H* plot exhibits a strong temperature‐dependent behavior and is attributed to a level crossing within the ferromagnetic state, which enables QTM once this excited state becomes thermally populated. Note that a broad peak centered at ~0.35 T can be ascribed to magnetization relaxation via a direct process [30].

Knowledge of the low‐lying electronic structure of the dimeric Dy^III^ complex enables a rational interpretation of its static and dynamic magnetic behavior. The single‐ion anisotropy, along with the local crystal field environment of the Dy^III^ ions, is responsible for the SMM behavior in complex **1** [2, 91]. However, at the single‐ion level, the magnetic characteristics of these systems would be dominated by QTM at zero field, as is often observed in hysteresis studies with a sharp drop of magnetization (butterfly‐shaped loops) [5, 6, 26]. In contrast, the antiferromagnetic interaction between the Dy^III^ pairs causes the zero‐field QTM process to shift away from zero field to higher fields, as evidenced by the S‐shaped µSQUID loops. Although the ground‐coupled state is nonmagnetic due to the antiferromagnetic interaction – hence, no SMM properties would be expected – the small separation (~1 cm^−1^) between the ground (nonmagnetic) and the first excited (ferromagnetic) exchange doublet leads to a considerable population of the latter even at 2 K, causing the response observed in the AC data.

### 2.6 Conclusions

In summary, we have synthesized and comprehensively characterized a new dinuclear Dy^III^‐based single‐molecule magnet with formula [Dy_2_(hynad)_2_(dbm)_4_] (**1**), supported by a bulky anionic N–O‐based bridging ligand. The coordination complex features a robust and nearly planar {Dy_2_(*µ*‐OR)_2_}^4+^ core enforced by two deprotonated *N*‐hydroxy‐1,8‐naphthalimide (hynadˉ) ligands, demonstrating that this anionic N–O bridging motif reliably stabilizes dimeric Dy^III^_2_ complexes in the presence of sterically demanding *β*‐diketonate coligands.

Magnetic studies establish zero‐field SMM behavior, with slow magnetic relaxation until 16 K, as evidenced by AC susceptibility measurements, and open hysteresis loops persisting up to 3.5 K. Analysis of temperature‐dependent relaxation times yields an effective energy barrier of *U*_eff_ = 119 cm^−1^ (~171 K). µSQUID measurements reveal a characteristic double‐S‐shaped hysteresis, consistent with an antiferromagnetically coupled ground state and a low‐lying ferromagnetic excited state responsible for the observed SMM behavior. *Ab initio* calculations confirm the highly axial nature of the two Dy^III^ ions, with ground KDs dominated by *m_J_* = ±15/2 character and the principal magnetic anisotropy axes oriented nearly perpendicular to the {Dy_2_(*µ*‐OR)_2_}^4+^ plane. The combined experimental and theoretical analysis shows that the intramolecular Dy···Dy interaction is weak and predominantly dipolar in origin, giving rise to an antiferromagnetic ground state separated by a small energy gap (~1 cm^−1^) from the first excited ferromagnetic exchange doublet. Thermal population of this excited state enables slow magnetic relaxation, with the Orbach process proceeding via higher‐lying exchange‐coupled states (~86 cm^−1^) that are consistent with the experimentally determined relaxation barrier.

Overall, this work highlights the effectiveness of anionic N–O‐based bridging ligands as versatile and underexplored building blocks for dinuclear lanthanide single‐molecule magnets. The hynadˉ ligand, in particular, provides a powerful structural platform that enables systematic tuning of peripheral coordination environments while maintaining favorable magnetic anisotropy and exchange characteristics. These findings expand the family of high‐performance and air‐stable Dy_2_ SMMs, paving the way for further development of N–O‐bridged molecular nanomagnets with enhanced magnetic bistability.

### 2.7 Experimental

#### 2.7.1 Synthesis

All manipulations were performed under aerobic conditions using materials (reagent grade) and solvents as received unless otherwise noted. The Dy(dbm)_3_·H_2_O starting material was synthesized following a similar reported procedure [75]. The identity and purity of the solid‐state product were confirmed by Fourier‐transform infrared (FT‐IR) spectroscopy and elemental analysis.

[Dy_2_(hynad)_2_(dbm)_4_]·DMF (**1**·DMF). To a stirred, orange colored solution of hynadH (0.10 mmol, 0.021 g) and Et_3_N (0.10 mmol, 0.014 mL) in a solvent mixture of CH_2_Cl_2_ and DMF (12 mL, 5:1, v/v), was added Dy(dbm)_3_·H_2_O (0.10 mmol, 0.079 g), resulting in a yellow‐colored solution. The solution was stirred for 15 min, then filtered, and the filtrate was left to evaporate slowly at room temperature. After 3 ‐ 4 days, X‐ray quality dark yellow block‐like crystals of **1**·DMF appeared. These were collected by filtration, washed with cold CH_2_Cl_2_ (2 × 2 mL) and Et_2_O (2 × 5 mL), and dried in air. Yield: 43 %. Anal. calc. (found) for C_87_H_63_Dy_2_N_3_O_15_ (**1**·DMF): C 60.91 (60.98), H 3.70 (3.79), N 2.45 (2.41). Selected FT‐IR data (cm^−1^): 3059 (w), 3026 (w), 1668 (s), 1644 (m), 1596 (s), 1550 (s), 1516 (s), 1477 (m), 1454 (s), 1388 (vs), 1284 (s), 1253 (m), 1221 (s), 1177 (w), 1157 (w), 1125 (w), 1071 (m), 1045 (s), 1023 (23), 997 (w), 908 (vs), 844 (s), 811 (w), 778 (s), 756 (m), 720 (s), 685 (s), 609 (m), 548 (w), 518 (s), 452 (w), 430 (w).

#### 2.7.2 Single Crystal X‐Ray Crystallography

Data for compound **1**·DMF were collected on a Rigaku XtaLAB Synergy‐S single‐crystal X‐ray diffractometer equipped with a CCD area detector and a graphite monochromator utilizing Cu K*α* radiation (*λ* = 1.54184 Å). A selected block‐like yellow crystal of **1**·DMF (0.232 × 0.186 × 0.128 mm) was attached to a glass fiber with paratone‐N oil and transferred to a goniostat for data collection. Empirical absorption corrections (multiscan based on symmetry‐related measurements) were applied using CrysAlis RED software [92]. The structures were solved by direct methods using SIR92 [93], and refined on *F* ^2^ using SHELXL97 [94], SHELXL‐2014/7 [95], and SHELXT [96]. Software packages used: CrysAlisCCD [92] for data collection, CrysAlisRED [92] for cell refinement and data reduction, and WINGX for geometric calculations [97]. All the non‐H‐atoms were treated anisotropically, whereas the H‐atoms were placed at calculated, ideal positions and refined as riding on their respective C‐atoms. Various figures of all the structures were created using Mercury [98] and Diamond [99] software packages.

#### 2.7.3 Physical Measurements

Elemental analyses (C, H, and N) were performed by the University of Patras microanalytical service. Infrared (IR) spectra (4000–400 cm^−1^) were recorded in the solid state using a Perkin–Elmer16 PC spectrometer with samples prepared as KBr pellets. Magnetic susceptibility measurements were conducted in a Quantum Design MPMS‐XL SQUID magnetometer on a polycrystalline sample of complex **1** in the temperature range of 2–300 K with an applied DC magnetic field of 1 kOe. The DC data were corrected for diamagnetic contributions from the sample holder and eicosane. Core diamagnetic corrections were performed employing Pascal's constants [100]. The AC data was collected in an oscillating magnetic field of 3.5 Oe and frequencies between 1 and 1.5 kHz. Low temperature (0.03–5 K) magnetization (*M*) versus field (*H*) studies were performed on single crystals of complex **1**·DMF employing a µSQUID array inside a dilution refrigerator equipped with a 3D vector magnet [101]. The high sensitivity of this magnetometer allows the study of single crystals of SMMs of the order 10–500 µm. The time resolution was approximately 1 ms. The magnetic field was applied in different directions of the µSQUID plane with a precision better than 0.1° by driving three orthogonal coils separately. To ensure good thermalization, each sample was fixed with Apiezon grease.

#### 2.7.4 *Ab Initio* Calculations

*Ab initio* calculations were performed on complex **1** using the CASSCF/SO‐RASSI/SINGLE_ANISO approach implemented in the OpenMolcas package [102, 103, 104, 105]. For the calculations, the crystal structure was employed without further optimizations, and the atoms were described using the standard basis sets from the ANO‐RCC library [106, 107, 108]. Due to the presence of two distinct Dy^III^ ions in the molecule, the dilution method was employed, where the Dy^III^ ion of interest was swapped with Y^III^ for the computations. Two calculations were conducted. A basis set of VTZP quality was employed for the Dy^III^ ions, while VDZP quality was employed for atoms directly bound to the Dy^III^ ions, and VDZ quality for the remaining atoms, using the second‐order DKH transformation [109]. Optimization of the molecular orbitals (MOs) was achieved by state‐averaged CASSCF calculations. The active space of Dy^III^ was defined by the nine 4f electrons in the seven 4f orbitals. Three calculations were carried out (RASSCF routine) with 21, 224, and 490 states for *S* = 5/2, *S* = 3/2, and *S* = 1/2, respectively. The CASSCF wavefunctions were subsequently mixed by spin‐orbit coupling, employing the RASSI routine [110, 111, 112] with all 21 states for *S* = 5/2 being included, while 128 and 130 states were included for *S* = 3/2 and *S* = 1/2. Lastly, the crystal field decomposition of the ground *J *= 15/2 multiplet of the ^6^H_15/2_ term was executed with the SINGLE_ANISO module.

## Conflicts of Interest

The authors declare no conflicts of interest.

## Supporting information




**Supporting File**: chem70936‐sup‐0001‐SuppMat.docx.
