# Supplementary material for: A High Energy Barrier DyIII 2 Single‐Molecule Magnet Supported by a Bulky, Anionic N–O Bridging Ligand
Source: Chemistry. 2026 Apr 1;32(31):e70936. doi: 10.1002/chem.70936 (PMC13485236; doi:10.1002/chem.70936)
Supplement: Supplementary file 1 [file CHEM-32-e70936-s001.docx]

**ELECTRONIC SUPPORTING INFORMATION**

**A High Energy Barrier Dy^III^_2_ Single-Molecule Magnet Supported by a Bulky, Anionic N–O Bridging Ligand**

Alexandros S. Armenis,^a^ Concepción Molina-Jirón,*^,b,c,d^ Konstantinos N. Pantelis,^a^ Wolfgang Wernsdorfer,^b,e^ Mario Ruben,^b,f^ Eufemio Moreno-Pineda,*^,b,g,h^ Theocharis C. Stamatatos*^,a^

^a^ Department of Chemistry, University of Patras, 265 04 Patras, Greece.

^b^ Institute of Quantum Materials and Technologies (IQMT), Karlsruhe Institute of Technology (KIT), Kaiserstraße 12, D-76131 Karlsruhe, Germany

^c^ Facultad de Ciencias Naturales, Exactas y Tecnología, Depto. de Bioquímica, Universidad de Panamá, Panamá 0824, Panama

^d^ Facultad de Ciencias Naturales, Exactas y Tecnología, Grupo de Investigación de Materiales, Universidad de Panamá, Panamá 0824, Panama

^e^ Physikalisches Institut, Karlsruhe Institute of Technology (KIT), Kaiserstraße 12, Karlsruhe, D-76131, Germany

^f^ Institute of Nanotechnology (INT), Karlsruhe Institute of Technology (KIT), Kaiserstraße 12, D-76131 Karlsruhe, Germany

^g^ Universidad de Panamá, Facultad de Ciencias Naturales, Exactas y Tecnología, Depto. de Química-Física, 0824 Panamá, Panamá.

^h^ Universidad de Panamá, Facultad de Ciencias Naturales, Exactas y Tecnología, Grupo de Investigación de Materiales, 0824 Panamá, Panamá.

Corresponding authors:

Prof. Dr. Theocharis C. Stamatatos – Dr. Eufemio Moreno-Pineda – Dr. Concepcion Molina-Jirón. E-mails: [thstama@upatras.gr](mailto:thstama@upatras.gr); [eufemio.moreno@up.ac.pa](mailto:eufemio.moreno@up.ac.pa); [concepcion.moreno@kit.edu](mailto:concepcion.moreno@kit.edu)

Table of Contents

[1. Crystallographic and structural details	2](#_Toc219618963)

[2. Magnetic studies	6](#_Toc219618964)

[3. Computational Details	11](#_Toc219618965)

# **1. Crystallographic and structural details**

**Table S1**. Crystal data and structural refinement parameters for complex **1**∙DMF.

| Parameter | **1**∙DMF |
| --- | --- |
| Formula | C_87_H_63_Dy_2_N_3_O_15_ |
| *F*_w_ / g∙mol^-1^ | 1715.41 |
| Crystal system | Trigonal |
| Space group | *R*3*c* |
| *a* / Å | 32.0339(2) |
| *b* / Å | 32.0339(2) |
| *c* / Å | 40.6528(2) |
| *α* / ° | 90 |
| *β* / ° | 90 |
| *γ* / ° | 120 |
| *V* / Å^3^ | 36127.7(5) |
| *Z* | 18 |
| *T* / *K* | 180.00(11) |
| Radiation / *μ* (mm^-1^) | Cu Kα / 1.54184 |
| *ρ*_calcd_ / g cm^-3^ | 1.419 |
| Reflections collected/unique (*R*_int_) | 41273/14144 (0.0234) |
| Reflections with *I*>2*σ*(*Ι*) | 13853 |
| No. of parameters | 977 |
| *R*_1_ [*I*>2*σ*(*Ι*)], *wR*_2_ [*I*>2*σ*(*Ι*)]*^a,b^* | 0.0335, 0.0885 |
| *R*_1_ (all data), *wR*_2_ (all data)*^a,b^* | 0.0343, 0.0890 |
| (Δ/*σ*)_max_ | 0.002 |
| Δ*ρ*_max_/Δ*ρ*_min_ (e Å^-3^) | 0.371 / −0.485 |
| CCDC number | 2523037 |

*^a^* *R*_1_ = Σ(|*F*_o_| - |*F*_o_|) / Σ(|*F*_o_|).

*^b^* *wR*_2_ = {Σ[*w*(*F*_o_^2^ - *F*_c_^2^)^2^] / Σ[*w*(*F*_o_^2^)^2^]}^1/2^, *w* = 1 / [*σ*^2^(*F*_o_^2^) + (*aP*)^2^ + *bP*], where *P* = [max(*F*_o_^2^, 0) + 2 *F*_c_^2^] / 3.

**Table S2.** Selected bond distances (Å) and angles (°) for complex **1**∙DMF.

| **Bond Distances (Å)** | | | | | |
| --- | --- | --- | --- | --- | --- |
| **Dy1** | | | **Dy2** | | |
| Dy1-O1  Dy1-O2  Dy1-O3  Dy1-O4  Dy1-O9  Dy1-O10  Dy1-O12  Dy1-O13 | 2.315(4)  2.321(5)  2.282(6)  2.277(4)  2.538(5)  2.348(6)  2.525(5)  2.339(6) | | Dy2-O5  Dy2-O6  Dy2-O7  Dy2-O8  Dy2-O10  Dy2-O11  Dy2-O13  Dy2-O14 | 2.323(5)  2.274(7)  2.238(9)  2.304(5)  2.350(8)  2.516(6)  2.369(8)  2.504(6) | |
| **Bond Angles (^o^)** | | | | | |
| **Dy1** | | | **Dy2** | | |
| Dy1-O10-Dy2 | | 118.5(1) | Dy2-O13-Dy1 | | 118.1(2) |
| O13-Dy1-O10 | | 61.9(2) | O13-Dy2-O10 | | 61.5(2) |
| O10-Dy1-O4 | | 86.6(2) | O8-Dy2-O13 | | 87.1(2) |
| O9-Dy1-O4 | | 77.1(2) | O8-Dy2-O14 | | 76.8(2) |
| O3-Dy1-O4 | | 74.9(2) | O8-Dy2-O11 | | 75.0(2) |
| O12-Dy1-O4 | | 75.5(2) | O8-Dy2-O7 | | 75.5(2) |
| O13-Dy1-O4 | | 94.0(2) | O8-Dy2-O10 | | 97.7(2) |
| O2-Dy1-O4 | | 136.9(2) | O8-Dy2-O6 | | 136.9(3) |
| O1-Dy1-O4 | | 148.2(2) | O8-Dy2-O5 | | 148.2(3) |
| O2-Dy1-O12 | | 67.9(2) | O6-Dy2-O11 | | 67.5(2) |
| O2-Dy1-O3 | | 83.7(2) | O6-Dy2-O10 | | 84.5(2) |
| O2-Dy1-O13 | | 89.7(2) | O6-Dy1-O7 | | 85.3(3) |
| O1-Dy1-O9 | | 71.4(2) | O5-Dy2-O14 | | 71.4(2) |
| O1-Dy1-O10 | | 76.8(2) | O5-Dy2-O13 | | 78.4(2) |
| O1-Dy1-O3 | | 100.7(1) | O5-Dy2-O7 | | 97.3(3) |

**Table S3.** Continuous Shape Measures (CShM) values for the potential coordination polyhedra of the 8-coordinate Dy^III^ centers in the structure of complex **1**∙DMF. The values in boldface indicate the closest polyhedron.

| Polyhedron*^a^* | **Dy1** | **Dy2** |
| --- | --- | --- |
| OP-8 | 33.00 | 32.79 |
| HPY-8 | 21.17 | 21.22 |
| HBPY-8 | 12.46 | 12.97 |
| CU-8 | 10.29 | 10.75 |
| SAPR-8 | 3.94 | 3.94 |
| **TDD-8** | **3.38** | **3.22** |
| JGBF-8 | 9.92 | 10.40 |
| JETBPY-8 | 21.64 | 21.78 |
| JBTPR-8 | 4.03 | 3.88 |
| BTPR-8 | 3.61 | 3.59 |
| JSD-8 | 4.03 | 4.07 |
| TT-8 | 11.09 | 11.52 |
| ETBPY-8 | 19.94 | 19.90 |

*^a^* Abbreviations: OP-8, octagon; HPY-8, heptagonal pyramid; HBPY-8, hexagonal bipyramid; CU-8, cube; SAPR-8, square antiprism; TDD-8, triangular dodecahedron; JGBF-8, Johnson gyrobifastigium; JETBPY-8, Johnson elongated triangular bipyramid; JBTPR-8, Johnson biaugmented trigonal prism; BTPR-8, biaugmented trigonal prism; JSD-8, Johnson snub diphenoid; TT-8, triakis tetrahedron; ETBPY-8, elongated trigonal bipyramid. The values in boldface indicate the closest polyhedron according to the Continuous Shape Measures.

**Figure S1**. (a) Representation of Dy_2_ complexes present in the unit cell of complex **1**∙DMF; (b) Supramolecular packing of the dinuclear Dy_2_ complexes in **1**·DMF viewed along the crystallographic *c*-axis, highlighting the cyclic arrangement of neighbouring units and the star-like motifs formed by inward-oriented phenyl rings of the dbmˉ ligands. Hydrogen atoms and lattice DMF solvate molecules are omitted for clarity. Colour code: Dy^III^, yellow; O, red; N, blue; C, dark grey.

**Figure S2**. A portion of the repeating dimers in the crystal of **1**∙DMF and their interactions with the neighbouring units through strong *π*–*π* stacking interactions (red dashed lines). The red spheres are the phenyl ring centroids. Hydrogen atoms and lattice DMF solvate molecules are omitted for clarity. Colour code: Dy^III^, yellow; O, red; N, blue; C, dark grey.

# **2. Magnetic studies**

**Figure S3**. (**A**) Temperature dependence of the in-phase (*χ*′_M_) *ac* magnetic susceptibility over the frequency range 0.1-1512 Hz, at zero *dc* field for complex **1**. (**B**) *χ*′_M_*T* vs *T* plots over the frequency range 0.1-1512 Hz at zero *dc* field for complex **1**. Solid lines represent the best fit to a generalised Debye model.

**Figure S4**. Frequency-dependence of the in-phase (*χ′*_M_) *ac* magnetic susceptibility over the temperature range 2-18 K, at zero *dc* field (**A**), and under an applied field of 750 Oe (**B**) for complex **1**. Solid lines represent the best fit to a generalised Debye model.

**Figure S5**. Temperature-dependence of the out-of-phase (*χ″*_M_) *ac* magnetic susceptibility over the frequency range 0.1-1512 Hz, at zero *dc* field (**A**), and under an applied field of 750 Oe (**B**) for complex **1**. Solid lines represent the best fit to a generalised Debye model.

**Figure S6**. Frequency-dependence of the in-phase (*χ′*_M_) (**A**) and out-of-phase (*χ″*_M_) (**B**) *ac* magnetic susceptibility over the frequency range 0.1-1512 Hz and for fields between 0 and 0.5 T at 4 K. Solid lines are fits to a generalised Debye model.

**Figure S7**. τ(*H*) behaviour of **1** at a fixed temperature of 4 K. The field at which relaxation is slower is found to be 75 mT (750 Oe). Moreover, the field at 180 mT coincides with the level crossing between the antiferromagnetic ground state and the ferromagnetic excited state, as also determined experimentally in the µSQUID loops and magnetic analysis.

**Table S4**. Relaxation fitting parameters in zero applied DC field for complex **1**.

| *T* / K | *χ*_S_ / cm^3^ mol^-1^ | *χ*_S_ (error) | *χ*_Τ_ / cm^3^ mol^-1^ | *χ*_T_ (error) | *τ* / s | *τ* (error) | *α* | *α* (error) |
| --- | --- | --- | --- | --- | --- | --- | --- | --- |
| 2 | 0.13292 | 0.01148 | 7.81879 | 0.08285 | 0.54854 | 0.01677 | 0.39402 | 0.00537 |
| 2.2 | 0.11771 | 0.01289 | 7.57954 | 0.08697 | 0.49734 | 0.01657 | 0.39583 | 0.006 |
| 2.4 | 0.11739 | 0.01276 | 7.31002 | 0.07998 | 0.44928 | 0.0142 | 0.39019 | 0.00599 |
| 2.6 | 0.10946 | 0.01301 | 7.04922 | 0.07513 | 0.40007 | 0.0123 | 0.38617 | 0.00613 |
| 2.8 | 0.10544 | 0.0175 | 6.57429 | 0.08456 | 0.31138 | 0.01138 | 0.36918 | 0.00831 |
| 3 | 0.10489 | 0.01381 | 6.52138 | 0.06493 | 0.29926 | 0.00846 | 0.36692 | 0.00655 |
| 3.2 | 0.10619 | 0.01549 | 6.2443 | 0.06409 | 0.24978 | 0.0072 | 0.35072 | 0.00738 |
| 3.6 | 0.11553 | 0.01923 | 5.69199 | 0.06063 | 0.16779 | 0.00496 | 0.31072 | 0.0093 |
| 4 | 0.12546 | 0.02174 | 5.20553 | 0.05342 | 0.11216 | 0.00325 | 0.27058 | 0.0107 |
| 4.4 | 0.12596 | 0.02236 | 4.79789 | 0.04483 | 0.07694 | 0.0021 | 0.23809 | 0.01122 |
| 4.8 | 0.12504 | 0.02214 | 4.45759 | 0.03771 | 0.05446 | 0.00141 | 0.21318 | 0.01133 |
| 5.2 | 0.12251 | 0.02148 | 4.169 | 0.032 | 0.03958 | 9.73937E-4 | 0.19479 | 0.0112 |
| 5.6 | 0.11949 | 0.02091 | 3.9199 | 0.02781 | 0.02945 | 6.9932E-4 | 0.18114 | 0.0111 |
| 6 | 0.11648 | 0.02098 | 3.70266 | 0.02527 | 0.02237 | 5.32374E-4 | 0.17133 | 0.01133 |
| 6.4 | 0.11312 | 0.02033 | 3.46152 | 0.02192 | 0.01621 | 3.7474E-4 | 0.16094 | 0.01121 |
| 7 | 0.1125 | 0.02059 | 3.25183 | 0.02009 | 0.01201 | 2.83093E-4 | 0.15413 | 0.01156 |
| 7.5 | 0.11379 | 0.02014 | 3.06587 | 0.01794 | 0.00909 | 2.11072E-4 | 0.14715 | 0.01151 |
| 8 | 0.11704 | 0.02087 | 2.9008 | 0.01704 | 0.00698 | 1.69503E-4 | 0.14327 | 0.01211 |
| 8.5 | 0.13479 | 0.02121 | 2.6247 | 0.0148 | 0.00435 | 1.09183E-4 | 0.13422 | 0.0127 |
| 9 | 0.14948 | 0.02109 | 2.49303 | 0.01357 | 0.00343 | 8.62361E-5 | 0.12732 | 0.01287 |
| 9.5 | 0.16178 | 0.01991 | 2.37376 | 0.01182 | 0.00271 | 6.46107E-5 | 0.11979 | 0.01234 |
| 10 | 0.17703 | 0.01997 | 2.26605 | 0.01091 | 0.00215 | 5.16658E-5 | 0.11471 | 0.01253 |
| 11 | 0.20058 | 0.01777 | 2.07733 | 0.00816 | 0.00137 | 2.90856E-5 | 0.1015 | 0.01129 |
| 12 | 0.2211 | 0.01637 | 1.91549 | 0.00619 | 8.68017E-4 | 1.67078E-5 | 0.08679 | 0.01036 |
| 13 | 0.2323 | 0.01234 | 1.78108 | 0.00369 | 5.45297E-4 | 7.65134E-6 | 0.07408 | 0.00749 |
| 14 | 0.22523 | 0.0161 | 1.66559 | 0.00351 | 3.41425E-4 | 6.15298E-6 | 0.07746 | 0.00884 |
| 15 | 0.24434 | 0.01561 | 1.56357 | 0.00239 | 2.19157E-4 | 3.86645E-6 | 0.06754 | 0.00774 |
| 16 | 0.25435 | 0.01968 | 1.47186 | 0.00186 | 1.39163E-4 | 3.26887E-6 | 0.06902 | 0.00821 |
| 17 | 0.32633 | 0.03441 | 1.3905 | 0.00206 | 9.6483E-5 | 4.34723E-6 | 0.05137 | 0.013 |
| 18 | 0.33201 | 0.07444 | 1.31817 | 0.00232 | 6.25324E-5 | 6.64804E-6 | 0.06345 | 0.02173 |

**Table S5**. Selected structural and magnetic characteristics of the highest-performing dinuclear Dy^III^ zero-field SMMs supported by N–O-based bridging ligands.

| **Complex Formula** | **Bridging Ligand** | **Coordination Geometry** | ***U*_eff_ [K]** | **Ref.** |
| --- | --- | --- | --- | --- |
| [Dy_2_(hfac)_6_(PzNO)_2_] |  | Triangular Dodecahedron | 226.73 | [13h]  main text |
| [Dy_2_(hfac)_6_(PyNO-NO_2_)_2_] |  | Square Antiprism | 204(8) | [13c]  main text |
| [Dy_2_(dbm)_4_(hynad)_2_]·DMF |  | Triangular Dodecahedron | 171(1) | t.w. |
| [Dy_2_(hfac)_6_(PyNO)_2_] |  | Square Antiprism | 167(1) | [13k]  main text |
| [Dy_2_(DClQ)_6_(bpmo)_2_] |  | Triangular Dodecahedron | 159 | [13l]  main text |
| [Dy_2_(tta)_6_(4-mepyNO)_2_]·CHCl_3_ |  | Triangular Dodecahedron | 121 | [13p]  main text |
| [a] Abbreviations: hfacˉ: hexafluoroacetylacetonate, dbmˉ: dibenzoylmethanoate, DClQˉ: 5,7-dichloro-8-hydroxyquinolinate, ttaˉ: thenoyltrifluoroacetonate; PzNO: pyrazine-N-oxide, PyNO-NO_2_: 4-Nitropyridine-N-oxide, hynadˉ: N-hydroxy-1,8-phthalimide, pyNO: pyridine-N-oxide, bpmo: 4,4′-bipyridine-N,N′-dioxide, 4-mepyNO: 4-methylpyridine-N-oxide, t.w.: this work. | | | | |

**Table S6**. Relaxation fitting parameters under an applied DC field of 750 Oe for complex **1**.

| *T* / K | *χ*_S_ / cm^3^ mol^-1^ | *χ*_S_ (error) | *χ*_Τ_ / cm^3^ mol^-1^ | *χ*_T_ (error) | *τ* / s | *τ* (error) | *α* | *α* (error) |
| --- | --- | --- | --- | --- | --- | --- | --- | --- |
| 2 | 0.08504 | 0.00771 | 8.53359 | 0.26555 | 4.28045 | 0.4222 | 0.49185 | 0.00597 |
| 2.2 | 0.07567 | 0.00854 | 8.18779 | 0.24807 | 3.45422 | 0.33374 | 0.4888 | 0.00637 |
| 2.4 | 0.08086 | 0.00666 | 7.59553 | 0.15299 | 2.50583 | 0.15862 | 0.47323 | 0.00492 |
| 2.6 | 0.07822 | 0.00596 | 7.28761 | 0.11381 | 1.9641 | 0.09558 | 0.46315 | 0.00427 |
| 2.8 | 0.06117 | 0.02083 | 7.17879 | 0.33162 | 1.56283 | 0.22714 | 0.46318 | 0.01383 |
| 3 | 0.07418 | 0.00643 | 6.95879 | 0.08807 | 1.27055 | 0.04902 | 0.44281 | 0.00425 |
| 3.2 | 0.07174 | 0.00755 | 6.83959 | 0.08658 | 1.00861 | 0.03833 | 0.4305 | 0.00474 |
| 3.6 | 0.08503 | 0.01434 | 6.14755 | 0.09732 | 0.50914 | 0.02269 | 0.38125 | 0.0084 |
| 4 | 0.10174 | 0.0219 | 5.44717 | 0.09007 | 0.25854 | 0.0115 | 0.32542 | 0.01229 |
| 4.4 | 0.10967 | 0.02522 | 4.91453 | 0.07133 | 0.14565 | 0.00576 | 0.2797 | 0.01388 |
| 4.8 | 0.11228 | 0.02607 | 4.51588 | 0.05637 | 0.08981 | 0.00321 | 0.24568 | 0.01427 |
| 5.2 | 0.11391 | 0.02564 | 4.1958 | 0.04532 | 0.05895 | 0.00193 | 0.22062 | 0.01408 |
| 5.6 | 0.11155 | 0.02539 | 3.92948 | 0.03832 | 0.04055 | 0.00127 | 0.20212 | 0.01405 |
| 6 | 0.11425 | 0.02463 | 3.70123 | 0.03266 | 0.02906 | 8.65824E-4 | 0.18708 | 0.01379 |
| 6.4 | 0.11053 | 0.02402 | 3.45572 | 0.02779 | 0.01987 | 5.7087E-4 | 0.1745 | 0.01361 |
| 7 | 0.11051 | 0.02363 | 3.24172 | 0.02433 | 0.01412 | 3.97964E-4 | 0.16455 | 0.01359 |
| 7.5 | 0.1143 | 0.02281 | 3.05435 | 0.0212 | 0.01037 | 2.82425E-4 | 0.15521 | 0.01333 |
| 8 | 0.11834 | 0.02274 | 2.88689 | 0.01924 | 0.00779 | 2.12305E-4 | 0.14851 | 0.01349 |
| 8.5 | 0.13597 | 0.0223 | 2.61053 | 0.01597 | 0.00471 | 1.27147E-4 | 0.13672 | 0.01361 |
| 9 | 0.14864 | 0.02239 | 2.47998 | 0.0147 | 0.00365 | 9.9361E-5 | 0.13092 | 0.01387 |
| 9.5 | 0.16405 | 0.02104 | 2.36085 | 0.01273 | 0.00286 | 7.35295E-5 | 0.122 | 0.01326 |
| 10 | 0.18493 | 0.01989 | 2.25252 | 0.01111 | 0.00227 | 5.53287E-5 | 0.11218 | 0.01277 |
| 11 | 0.19213 | 0.01913 | 2.15558 | 0.00975 | 0.00178 | 4.15243E-5 | 0.10748 | 0.0123 |
| 12 | 0.20758 | 0.01764 | 2.06584 | 0.00824 | 0.00142 | 3.04848E-5 | 0.10002 | 0.01144 |
| 13 | 0.22226 | 0.01471 | 1.90505 | 0.00562 | 8.87896E-4 | 1.55387E-5 | 0.08692 | 0.00942 |
| 14 | 0.23167 | 0.01392 | 1.77265 | 0.00424 | 5.61859E-4 | 8.99108E-6 | 0.07329 | 0.00859 |
| 15 | 0.23025 | 0.01573 | 1.65628 | 0.00352 | 3.50562E-4 | 6.21143E-6 | 0.07259 | 0.00885 |
| 16 | 0.24171 | 0.01505 | 1.55367 | 0.00233 | 2.21175E-4 | 3.77015E-6 | 0.06536 | 0.00755 |
| 17 | 0.2308 | 0.02165 | 1.46338 | 0.00202 | 1.37526E-4 | 3.51988E-6 | 0.07046 | 0.00885 |
| 18 | 0.29583 | 0.03617 | 1.3837 | 0.00205 | 9.31816E-5 | 4.39971E-6 | 0.0618 | 0.01298 |

# **3. Computational Details**

**Table S7.** Computed energy levels (the ground state is set at zero), composition of the *g*-tensor (*g*_x_, *g*_y_, *g*_z_), and the main components (>10%) of the wavefunction for each *m_J_* state of the ground-state multiplet ^6^H_15/2_ for the **Dy1** in complex **1**, at the CASSCF level.

| ***ab initio energy* (cm^–1^)** | ***g*_x_** | ***g*_y_** | ***g*_z_** | ***Wavefunction composition*** |
| --- | --- | --- | --- | --- |
| 0 | 0.05023 | 0.0874 | 19.7200 | 98.1%$\left\vert\left. \text{±15/2} \right\rangle\right.$ |
| 118.3 | 0.5493 | 0.7277 | 18.5767 | 11%$\left\vert\left. \text{±7/2} \right\rangle\right.$+ 17.8%$\left\vert\left. \text{±5/2} \right\rangle\right.$+ 23.5%$\left\vert\left. \text{±3/2} \right\rangle\right.$+ 28.6%$\left\vert\left. \text{±1/2} \right\rangle\right.$ |
| 180.0 | 0.5529 | 2.1729 | 14.6164 | 71.8%$\left\vert\left. \text{±13/2} \right\rangle\right.$ |
| 257.6 | 3.6233 | 4.6364 | 10.8396 | 41.4%$\left\vert\left. \text{±11/2} \right\rangle\right.$+ 22.8%$\left\vert\left. \text{±9/2} \right\rangle\right.$+ 10.4%$\left\vert\left. \text{±5/2} \right\rangle\right.$ |
| 321.6 | 2.1927 | 4.5510 | 11.2214 | 10.3%$\left\vert\left. \text{±11/2} \right\rangle\right.$+ 13.8%$\left\vert\left. \text{±9/2} \right\rangle\right.$+ 35.5%$\left\vert\left. \text{±7/2} \right\rangle\right.$+ 10%$\left\vert\left. \text{±5/2} \right\rangle\right.$ + 18.7%$\left\vert\left. \text{±1/2} \right\rangle\right.$ |
| 382.4 | 0.4875 | 2.3889 | 13.6928 | 12.7%$\left\vert\left. \text{±9/2} \right\rangle\right.$+ 34.3%$\left\vert\left. \text{±5/2} \right\rangle\right.$+ 28.7%$\left\vert\left. \text{±3/2} \right\rangle\right.$ |
| 461.4 | 0.8853 | 1.0373 | 17.3947 | 23.4%$\left\vert\left. \text{±11/2} \right\rangle\right.$+ 30.5%$\left\vert\left. \text{±9/2} \right\rangle\right.$+ 21.8%$\left\vert\left. \text{±7/2} \right\rangle\right.$ + 11.8%$\left\vert\left. \text{±1/2} \right\rangle\right.$ |
| 564.0 | 0.1008 | 0.1880 | 19.2227 | 13.5%$\left\vert\left. \text{±7/2} \right\rangle\right.$+ 20.7%$\left\vert\left. \text{±5/2} \right\rangle\right.$+ 26.6%$\left\vert\left. \text{±3/2} \right\rangle\right.$ + 29.4%$\left\vert\left. \text{±1/2} \right\rangle\right.$ |

**Table S8.** Computed energy levels (the ground state is set at zero), composition of the *g*-tensor (*g*_x_, *g*_y_, *g*_z_), and the main components (>10%) of the wavefunction for each *m_J_* state of the ground-state multiplet ^6^H_15/2_ for the **Dy2** in complex **1**, at the CASSCF level.

| ***ab initio energy* (cm^–1^)** | ***g_x_*** | ***g_y_*** | ***g_z_*** | ***Wavefunction*** |
| --- | --- | --- | --- | --- |
| 0 | 0.0373 | 0.1027 | 19.7031 | 98.4%$\left\vert\left. \text{±15/2} \right\rangle\right.$ |
| 84.9 | 0.1654 | 0.2488 | 19.2583 | 10.9%$\left\vert\left. \text{±7/2} \right\rangle\right.$+ 18.8%$\left\vert\left. \text{±5/2} \right\rangle\right.$+ 25.7%$\left\vert\left. \text{±3/2} \right\rangle\right.$+ 31.3%$\left\vert\left. \text{±1/2} \right\rangle\right.$ |
| 172.1 | 0.08523 | 0.4548 | 16.0085 | 70.9%$\left\vert\left. \text{±13/2} \right\rangle\right.$ + 15.1%$\left\vert\left. \text{±11/2} \right\rangle\right.$ |
| 227.3 | 2.9932 | 3.9295 | 12.3300 | 11.7%$\left\vert\left. \text{±13/2} \right\rangle\right.$+ 24.8%$\left\vert\left. \text{±11/2} \right\rangle\right.$+ 39.8%$\left\vert\left. \text{±9/2} \right\rangle\right.$ |
| 279.8 | 2.9361 | 4.8281 | 4.8281 | 20.7%$\left\vert\left. \text{±11/2} \right\rangle\right.$+ 41.7%$\left\vert\left. \text{±7/2} \right\rangle\right.$+ 18.5%$\left\vert\left. \text{±5/2} \right\rangle\right.$ |
| 368.6 | 0.3392 | 3.4959 | 13.1675 | 10.1%$\left\vert\left. \text{±9/2} \right\rangle\right.$+ 34.8%$\left\vert\left. \text{±5/2} \right\rangle\right.$+ 30.2%$\left\vert\left. \text{±3/2} \right\rangle\right.$ |
| 443.4 | 1.2381 | 2.3027 | 14.4192 | 23.8%$\left\vert\left. \text{±11/2} \right\rangle\right.$+ 27.7%$\left\vert\left. \text{±9/2} \right\rangle\right.$+ 18.8%$\left\vert\left. \text{±7/2} \right\rangle\right.$ + 18.2%$\left\vert\left. \text{±1/2} \right\rangle\right.$ |
| 498.1 | 0.5665 | 1.9123 | 17.8239 | 10.7%$\left\vert\left. \text{±7/2} \right\rangle\right.$+ 17.3%$\left\vert\left. \text{±5/2} \right\rangle\right.$+ 27.4%$\left\vert\left. \text{±3/2} \right\rangle\right.$+ 30.9%$\left\vert\left. \text{±1/2} \right\rangle\right.$ |

**Table S9.** Crystal field Hamiltonian is given as $\hat{H}_{CF}=\sum_{k,q} B_{k}^{q}O_{k}^{q}$, and the extended Stevens operator coefficients $B_{k}^{q}$ are extracted from CASSCF calculations for complex **1.**

|  |  | $B_{k}^{q}$ | |
| --- | --- | --- | --- |
| *k* | *q* | **Dy1** | **Dy2** |
| 2 | -2 | 0.74857 | -0.07839 |
| 2 | -1 | 1.60343 | 2.41094 |
| 2 | 0 | -1.77807 | -1.52953 |
| 2 | 1 | 1.23361 | 1.62158 |
| 2 | 2 | 2.76909 | 2.29294 |
| 4 | -4 | 0.0099 | 0.0085 |
| 4 | -3 | -0.03102 | -0.03099 |
| 4 | -2 | 0.01774 | 0.01638 |
| 4 | -1 | 0.00392 | 7.44397E-4 |
| 4 | 0 | -0.00535 | -0.00511 |
| 4 | 1 | -0.0088 | -0.00866 |
| 4 | 2 | -0.01639 | -0.02096 |
| 4 | 3 | -0.02664 | -0.02683 |
| 4 | 4 | -0.00521 | -0.01288 |
| 6 | -6 | 9.02266E-5 | 1.7518E-4 |
| 6 | -5 | -7.54207E-4 | -6.933E-4 |
| 6 | -4 | -1.85212E-5 | -4.51994E-6 |
| 6 | -3 | -6.22609E-5 | -6.21827E-5 |
| 6 | -2 | -9.5994E-5 | -1.00976E-4 |
| 6 | -1 | -2.38392E-4 | -2.44761E-4 |
| 6 | 0 | -7.30498E-6 | -1.23858E-5 |
| 6 | 1 | 6.79294E-6 | -3.78131E-5 |
| 6 | 2 | 5.77993E-5 | 5.88025E-5 |
| 6 | 3 | -4.08384E-5 | -6.70968E-5 |
| 6 | 4 | -3.32829E-5 | -3.62159E-5 |
| 6 | 5 | -4.12037E-5 | -1.58036E-4 |
| 6 | 6 | 3.91669E-4 | 3.52181E-4 |

4. **Dipolar Matrix**

Dipolar interactions between the Dy(III) ions were calculated using an in‑house Python routine that constructs the full anisotropic dipole–dipole coupling tensor, making use of the local magnetic orientations obtained from the *ab initio* SINGLE_ANISO calculations. For each Dy(III) center, the magnetic anisotropy was treated explicitly by assigning principal values from CASSCF, together with the corresponding unit vectors defining the local magnetic coordinate system. These vectors were used to build a right‑handed orthonormal rotation matrix, which subsequently transformed the principal‑axis g tensor into the laboratory reference frame.

The dipolar couplings between the ions were calculated from the standard Hamiltonian:

$H_{dip}= {\mathbf{-}2\mathbf{S}}_{i}^{T}\mathbf{D}_{ij}\mathbf{S}_{j}$,

where the full dipolar tensor $\mathbf{D}_{ij}$ is given by

$$\mathbf{D}_{ij}=\frac{{\mu_{0}\mu}_{B}^{2}}{4\pi r_{ij}^{3}}-\left[ \mathbf{g}_{i}^{T}.\mathbf{g}_{j}-3\left( \mathbf{g}_{i}^{T}.{\hat{\mathbf{r}}}_{ij} \right).\left( \mathbf{g}_{i}^{T}.{\hat{\mathbf{r}}}_{ij} \right)^{T} \right]$$

Here $r_{ij}$ is the Dy–Dy separation, and ${\hat{\mathbf{r}}}_{ij}$ is the corresponding unit vector.

The obtained dipolar matrix for Dy(1)⋅⋅⋅Dy(2) has the form (for an -2*J* Hamiltonian):

$\mathbf{D}_{1-2'}= \left( \begin{matrix} -0.109385 & -0.264661 & 0.192809 \\ -0.320729 & -0.775522 & 0.565003 \\ 0.18784 & 0.454208 & -0.330919 \end{matrix} \right)$ cm^–1^

These dipolar interaction values can be projected into an m*_J_* = 15/2 state by multiplication by a 1/225 factor. The strongest component of the calculated dipolar coupling, projected onto a m*_J_* = 15/2 manifold) is -3.4×10^-3^ cm^-1^ (5 mK), which is of the same order as the exchange value from µSQUID studies.

Calculation of the dipolar interaction for the nearest intermolecular Dy(III) centre at a 9.067(5) Å distance leads to the strongest component to be *J*_inter_ (*S*_eff_ = ½) -0.095 cm^-1^ (-13 mK), which projected onto a m*_J_* = 15/2 state would be -4.2×10^-4^ cm^-1^ (-0.6 mK).
